# Epidemiology of and Risk Factors for COVID-19 Infection among Health Care Workers: A Multi-Centre Comparative Study

**DOI:** 10.3390/ijerph17197149

**Published:** 2020-09-29

**Authors:** Jia-Te Wei, Zhi-Dong Liu, Zheng-Wei Fan, Lin Zhao, Wu-Chun Cao

**Affiliations:** 1 Institute of EcoHealth, School of Public Health, Cheeloo College of Medicine, Shandong University, Jinan 250012, Shandong, China; 201614261@mail.sdu.edu.cn (J.-T.W.); liuzhidong@mail.sdu.edu.cn (Z.-D.L.); 2 State Key Laboratory of Pathogen and Biosecurity, Beijing Institute of Microbiology and Epidemiology, Beijing 100071, China; zwfan9128@163.com

**Keywords:** COVID-19, health care worker, epidemiology, attack rate, risk factor

## Abstract

Healthcare workers (HCWs) worldwide are putting themselves at high risks of coronavirus disease 2019 (COVID-19) by treating a large number of patients while lacking protective equipment. We aim to provide a scientific basis for preventing and controlling the COVID-19 infection among HCWs. We used data on COVID-19 cases in the city of Wuhan to compare epidemiological characteristics between HCWs and non-HCWs and explored the risk factors for infection and deterioration among HCWs based on hospital settings. The attack rate (AR) of HCWs in the hospital can reach up to 11.9% in Wuhan. The time interval from symptom onset to diagnosis in HCWs and non-HCWs dropped rapidly over time. From mid-January, the median time interval of HCW cases was significantly shorter than in non-HCW cases. Cases of HCWs and non-HCWs both clustered in northwestern urban districts rather than in rural districts. HCWs working in county-level hospitals in high-risk areas were more vulnerable to COVID-19. HCW cases working in general, ophthalmology, and respiratory departments were prone to deteriorate compared with cases working in the infection department. The AR of COVID-19 in HCWs are higher than in non-HCWs. Multiple factors in hospital settings may play important roles in the transmission of COVID-19. Effective measures should be enhanced to prevent HCWs from COVID-19 infection.

## 1. Introduction

The outbreak of coronavirus disease 2019 (COVID-19), caused by severe acute respiratory syndrome coronavirus 2 (SARS-CoV-2), was first reported in Wuhan, China, in December 2019 [1]. In a short period, COVID-19 has spread fast worldwide, posing severe threats to global health. On 11 March 2020, the World Health Organization (WHO) declared the outbreak as a pandemic [2]. To contain this pandemic, many measures have been taken such as social distancing, wearing facemasks, and even city lockdowns [3]. Unfortunately, varying degrees of implements in different countries have not effectively stopped the global spread of COVID-19. So far, over 30 million people from over 180 countries have been affected, with a fatality rate of around 3.0% [4].

During this pandemic, healthcare workers (HCWs) are undoubtedly among the most suffering, facing an overwhelming number patients and lacking protective resources [5]. The infection of COVID-19 among HCWs has become a common phenomenon, especially in hospitals in the epicenter. During the past outbreak of COVID-19 in mainland China, over 3000 HCWs were infected, most of which came from the epicenter in Wuhan [6]. Moreover, infected HCWs and patients in hospital settings could play an important role in facilitating transmission and enlarging spread [7]. Understanding epidemic characteristics and risk factors of COVID-19 infection in hospitals, especially among HCWs, are indispensable for the prevention and control of COVID-19.

To our best knowledge, most published studies focusing on COVID-19 infection among HCWs are single-centered, limited in comparing the infection in HCWs and non-HCWs [8]. In this multi-center cross-sectional study, we utilized the data containing all confirmed COVID-19 cases in Wuhan during the outbreak, thoroughly compared epidemic characteristics between HCWs and non-HCWs, and explored the risk factors for infection and deterioration among HCWs based on hospital settings. Through this study, we hope to provide science-based evidence for infection prevention and control in hospital settings.

## 2. Materials and Methods

### 2.1. Data Sources

We collected data of confirmed COVID-19 cases in Wuhan from the National Notifiable Infectious Disease Information System until 27 February 2020. The database contains detailed anonymous information for confirmed cases, including age, sex, residence location, occupation, date of illness onset, date of diagnosis, date of death (if applicable), clinical classification, reported hospital, and department in which the infected HCWs work (if applicable). The list of designated hospitals for treating COVID-19 in Wuhan was obtained from the official website of the Health Commission of Hubei Province (http://wjw.hubei.gov.cn/bmdt/ztzl/fkxxgzbdgrfyyq/fkdt/202002/t20200222_2145016.shtml). Data on the total number of HCWs, nurses and hospital beds, classification (provincial, municipal, and county-level), and type (general, special, and Chinese medical) of 36 main hospitals in Wuhan was extracted from the Wuhan Health Statistical Yearbook (https://data.cnki.net/trade/Yearbook/Single/N2020010198?z=Z020). Data on the permanent population in every county of Wuhan were extracted from the official website of the Wuhan Bureau of Statistics (http://tjj.wuhan.gov.cn/). As this study constituted data analysis rather than research in human beings, ethical approval from institutional review boards was not required.

### 2.2. Case Definition of COVID-19

All COVID-19 cases in our study were confirmed according to the Guideline for Diagnosis and Treatment of Novel Coronavirus Pneumonia issued by the National Health Commission (NHC) (http://www.gov.cn/zhuanti/2020-02/09/5476407/files/765d1e65b7d1443081053c29ad37fb07.pdf). The confirmed cases were patients who had related epidemiological history and clinical manifestations with one of the following etiological evidences: SARS-CoV-2 nucleic acid detected by specific real-time PCR assay or viral gene sequence homologous to SARS-CoV-2. Confirmed cases were classified into four types, including mild cases, moderate cases, severe cases, and critical cases, according to the severity of their symptoms.

### 2.3. Statistical Analysis

To compare the basic characteristics of HCWs and non-HCWs, we calculated the attack rate (AR) of HCWs or non-HCWs in the city of Wuhan at first, dividing the cumulative number of cases by the total number of HCWs or non-HCWs. Then, the AR in a unit was calculated. For HCWs, the unit was defined as the hospital reporting the HCW cases; for non-HCWs, the unit was defined as a county (i.e., lower level of an administrative region than a city). The proportion of severe and critical cases (PSCC) among total confirmed cases of HCWs or non-HCWs was evaluated. The case fatality rate (CFR) was also estimated as the percentage of cumulative death numbers divided by the total number of infections of HCWs or non-HCWs. Descriptive statistics (median and interquartile range, IQR, for skewed continuous data and percentages for categorical data) were used to report the basic characteristics of confirmed cases. Kruskal–Wallis and chi-square tests were used for comparison of continuous variables and categorical variables, respectively. To visualize the spatial distribution of HCW and non-HCW cases, we geolocated the sites of confirmed cases on the map of Wuhan using ArcGIS 10.2 (Esri, RedLands, CA, USA). A retrospective space-time permutation model in SaTScan 9.6 (Martin Kulldorff, Boston, MA, USA) was used to identify COVID-19 clusters of HCWs and non-HCWs [9].

Using the data on 36 main hospitals in Wuhan, we conducted multivariate linear regression to evaluate the impacts of having fever clinic or not, level of the hospital (county-level, municipal, or provincial), type of hospital (special, general, or Chinese medical hospital), nurse/bed ratio, and the county where the hospital was located on AR of main hospitals. For hospitals reporting the department distribution of HCW cases (five general hospitals with 324 confirmed HCW cases), backward stepwise multivariate logistic regression was utilized to identify high-risk departments where HCW cases worked to develop severe or critical symptoms. Variance inflation factor (VIF) was also calculated to check for collinearity with the threshold of 10. Modelling processes were conducted with R 3.6.1 (The R Core Team, R Foundation for Statistical Computing, Vienna, Austria). All estimation of probabilities (*p*-value) were two-sided, and *p*-value < 0.05 was statistically significant.

## 3. Results

### 3.1. General Characteristics of HCW and Non-HCW Cases

As of 27 February 2020, a total of 48,313 COVID-19 cases including 2463 (5.1%) HCWs and 45,850 (94.9%) non-HCWs were confirmed in Wuhan. The confirmed HCW cases were distributed in 100 hospitals, and 79.0% of them came from 36 main hospitals in Wuhan. In 48 designated hospitals for treating COVID-19 in Wuhan, 32 (67.7%) reported HCW cases. In general, the AR of HCWs was around four times higher than non-HCWs in Wuhan, and the value reached up to 11.9% in one of the main hospitals. However, the PSCC and CFR of HCW cases were significantly lower than non-HCWs (*p*-value < 0.001). In addition, 71.5% of HCW cases were female, higher than that in non-HCW cases. The median age of HCW cases was 36 years old, significantly younger than that of non-HCW cases (Table 1).

### 3.2. Time from Symptom Onset to Diagnosis of HCW and Non-HCW Cases

**Figure 1:**
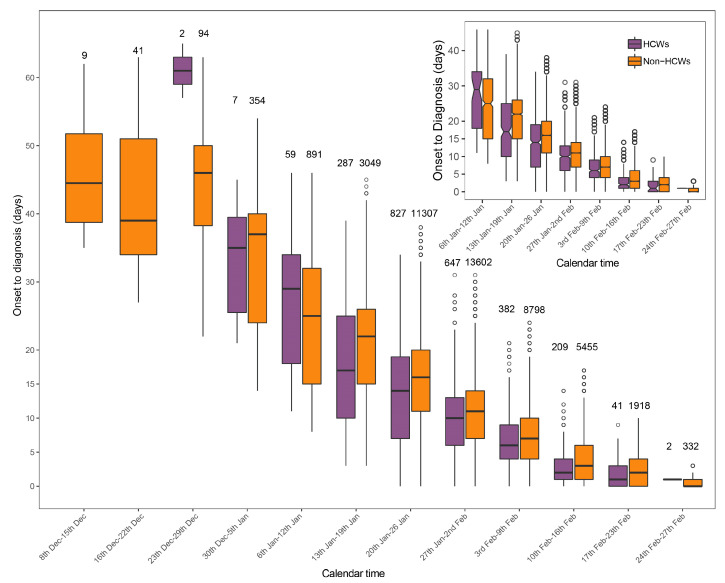
Time interval from symptom onset to diagnosis against calendar time among healthcare workers (HCWs) and non-HCW cases of COVID-19 in Wuhan. The case number in each group was shown over each box in the main figure. From 6 January 2020, the box plot was shown with notches in smaller scale, which represent the 95% confidence intervals of medians of time from symptom onset to diagnosis.

In general, there was no significant difference in days from symptom onset to diagnosis between HCW and non-HCW cases, both with the median (IQR) of 10 (5, 16) (Table 1). To further explore the difference, we stratified the days from symptom onset to diagnosis into several groups by the week after the first confirmed case shown (8 December 2019). The symptom onset date of the first HCW cases was three weeks later than non-HCW cases, with the longest interval from symptom onset to diagnosis (65 days) among total confirmed cases. The time interval of both HCWs and non-HCWs dropped rapidly over time, and from the sixth week after the first confirmed case (mid-January), the median time from onset to diagnosis of HCW cases was significantly shorter than non-HCW cases (Figure 1).

### 3.3. Spatial-Temporal Distributions of HCW and Non-HCW Cases

**Figure 2:**
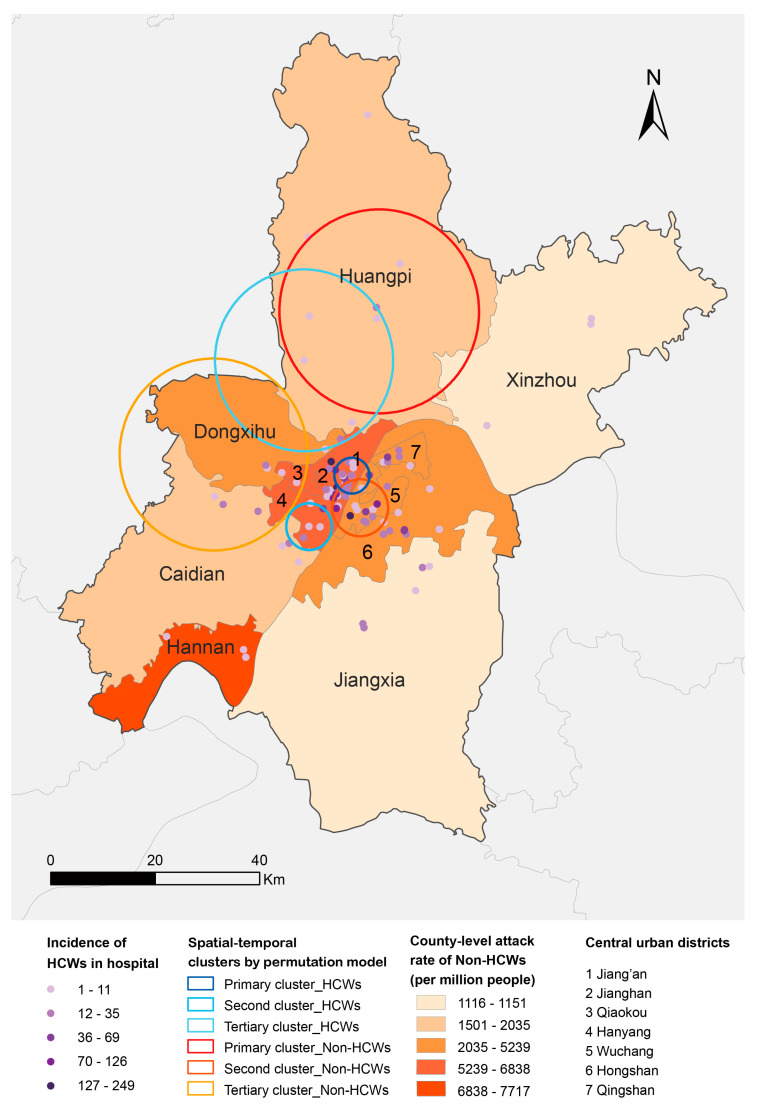
Distributions with spatial-temporal clusters of healthcare workers (HCWs) and non-HCW cases of COVID-19 in Wuhan. For HCWs, the number of cases was counted in each affected hospital, for non-HCWs, the attack rate was calculated in each county. Spatial-temporal clusters were identified by permutation model in HCW and non-HCW cases.

We geolocated the hospitals reporting HCW cases and residential locations of non-HCW cases, visualizing the case distributions in Wuhan. Most (70.0%) affected hospitals were located in central urban areas, with up to 249 HCWs infected by COVID-19 in a hospital. There were also large spatial differences of ARs in non-HCWs, ranging from 1116 to 7717 per million people. The results of the space-time permutation model showed that spatial distribution patterns were similar between HCW and non-HCW cases, both mainly clustered in northwestern urban districts than in rural districts (Figure 2). HCW cases in 36 main hospitals mainly distributed from mid-January to mid-February, with no case found before 23 December 2019 and after 23 February 2020 (Table 2).

### 3.4. Risk Factors of HCWs for Infection and Deterioration

We conducted multivariate linear regression to evaluate the effects of hospital characteristics on COVID-19 infection of HCWs. We found that, compared to the provincial hospital, the AR in the county-level hospital was significantly higher, with the RR of 1.04 (95% CI 1.01–1.07). The hospitals located in Hongshan (RR = 1.08, 95% CI 1.02–1.14), Wuchang (RR = 1.08, 95% CI 1.03–1.14), Hanyang (RR = 1.08, 95% CI 1.01–1.15), and Jiang’an (RR = 1.10, 95% CI 1.03–1.16) had significantly higher ARs of HCWs than hospitals in Xinzhou, where the least non-HCW cases were reported (Table 3). For five general hospitals reporting the department distribution of 324 HCW cases, the results of multivariate logistic regression showed that after adjustment of age, sex and days from symptom onset to diagnosis, HCWs worked in the general department (OR = 2.86, 95% CI 1.20–6.66), ophthalmology department (OR = 4.45, 95% CI 1.88–10.44), and respiratory department (OR = 13.35, 95% CI 3.93–47.23) were more likely to develop severe and critical symptoms than HCWs cases working in the infection department (Table 4). Estimated VIFs in the above models were all smaller than 10, indicating no collinearity existed among variables we explored in either model. 

## 4. Discussion

The shock of the COVID-19 pandemic to the world has extremely challenged the capacity of the global public health care system. The epidemiology of HCWs has been summarized in several studies all over the world [10,11,12,13]. In this study, we did not generally describe the infection status of HCWs but tried to compare multiple epidemiological characteristics with non-HCWs. Furthermore, we tried to identify underlying factors for COVID-19 transmission and deterioration based on hospital settings, thus providing a scientific basis for prevention and control policies.

In this outbreak of COVID-19, the proportion of HCWs among total cases was significantly lower than the 2003 outbreak of SARS in mainland China [14]. However, the AR in HCWs was around four times higher than non-HCWs in Wuhan, and when it comes to individual hospitals, the value of AR could reach 11.9%. Previous studies in Wuhan have reported consistently that the AR in HCWs was significantly higher than in non-HCWs, but no study has thoroughly compared ARs in different individual hospitals [11,15]. The results on attack rates of HCWs in foreign hospitals varied a lot with countries, severity of epidemic and study design, from 0.4% to 29.1% [8]. To effectively control the outbreak, 48 hospitals in Wuhan were designated by the government to treat infected patients in severe and critical conditions. Notably, more than half (67.7%) of them have reported HCW cases. This information indicates that, on the one hand, the risk of designated hospitals being affected was high; on the other hand, a part of the hospitals had no infected HCW thanks to the strict occupational guidance and adequate personal protective equipment (PPE) [16,17]. Despite higher AR, the lower PSCC and CFR in HCW cases may be due to the convenience for healthcare resources or better health awareness. A higher proportion of females and younger age in HCW cases may be because of the composition of HCWs, in that more young people and females serve as HCWs (especially nurses) with longer contacting hours to COVID-19 patients and higher exposed risks [11,18].

The time intervals from symptom onset to diagnosis of HCW and non-HCW cases were generally consistent, with median (IQR) valued 10 (5, 16). Some studies on the radiology of COVID-19 have discovered that more consolidated lung lesion would appear after five days from disease onset, and the lesion would be most severe in 10 days [19,20]. Therefore, almost half of the cases might have severely impaired lung function at the time of diagnosis, increasing the difficulty of disease treatment. Fortunately, the interval from onset to diagnosis of HCW and non-HCW cases both decreased rapidly to less than 10 days in February 2020. From mid-January, the median time interval of HCW cases became significantly shorter than non-HCW cases, suggesting HCWs might concentrate more on their health status than non-HCWs and tended to be tested as soon as possible when suspected symptoms arose.

We thoroughly characterized the spatial-temporal distributions of HCW and non-HCW cases, which have not been conducted by previous studies. From the map on the distribution of HCW and non-HCW cases, we found two groups of people had similar spatial distribution patterns that mainly clustered in northwestern urban districts. This similarity shows that high-risk areas for non-HCWs could also be high-risk for HCWs. We also noticed that some hospitals in high-risk areas had low incidences among HCWs, indicating the crucial role of PPE. Improper PPE was observed to increase 2.82 times of risk for HCWs infection [21]. The HCW cases most distributed from mid-January to mid-February, which was shorter than the COVID-19 outbreak in non-HCWs. This may attribute to the centralized quarantine and treatment started from 2 February 2020 [22].

Through modelling, we identified the high-risk type of hospitals and departments for COVID-19 infection. HCWs working in county-level hospitals in areas with more non-HCW cases were more vulnerable to COVID-19. Another study in Wuhan reported that general hospitals had significantly higher HCW infections of COVID-19 [11]. Most (72.2%) main hospitals in Wuhan are general hospitals owning multiple departments. Low-grade general hospitals may not be as well-equipped for protective guidance and facilities as high-grade general hospitals, which may have caused more HCW cases, especially in the areas with more non-HCWs. Surprisingly, HCW cases working in general, ophthalmology, and respiratory departments were easier to develop the symptoms into severe and critical conditions compared with HCW cases working in the infection department. Non-first-line HCWs were reported to have a significantly higher AR than first-line HCWs [15], but no study has yet explored the high-risk departments for COVID-19 deterioration. HCW cases working in the infection department were most from the fever clinic in our study, who have a better awareness of being infected than general HCWs once the suspecting symptoms attack them. The deterioration risk of HCWs working in the ophthalmology department was higher than the infection department and other general departments, which may be because of its special diagnosis and treatment require HCWs to be very close to the face of patients, as well as poor PPE and lack of drug supply [23]. The deterioration risk of HCWs working in the respiratory department was the highest, which may due to the shortage of HCWs and PPE at the beginning of the outbreak causing prolonged hours to care for the COVID-19 patients and lack of chance to treat the disease [21,24].

This study has several limitations. First, we can only get the data of the hospitals reporting the HCW cases, which were not necessarily the hospitals where HCWs worked and got infected. However, according to the regulations that suspected or confirmed cases should be reported within two hours by the hospital they visited, assuming that these hospitals were their workplaces would be rational. Second, we compared the ARs of HCWs and non-HCWs without adjusting for the demographic characteristics such as age and sex; thus, the results should be interpreted with caution. Finally, the characteristics of asymptomatic cases in HCWs and non-HCWs were not discussed due to the lack of data, and the differences in detecting modes among different hospitals may bias the results of our study.

## 5. Conclusions

COVID-19 infection in HCWs is higher than non-HCWs. Lower-grade general hospitals in high-risk areas should pay more attention to policies and measures preventing nosocomial transmission. Despite infection and respiratory departments, HCWs in general departments (especially in the ophthalmology department) should also be alert to COVID-19 and well-protected. Other measures should be taken, including adequate nutrition, job rotation, and psychological support to prevent HCWs from contracting COVID-19.

## Figures and Tables

**Table 1:** Comparison of basic characteristics between healthcare workers (HCWs) and non-HCWs in Wuhan.

	HCWs	Non-HCWs	*p*-Value
Demographic characteristics			
Male	703	22326	<0.001 ^a,^*
Female	1760	23524	
Female (%)	71.5	51.3	
Age (median [IQR])	36 (29, 44)	56 (44, 66)	<0.001 ^b,^*
Days from onset to diagnosis (median [IQR])	10 (5, 16)	10 (5, 16)	0.68 ^b^
Infection status			
Non-infected individual	107126	10925561	<0.001 ^a,^*
Infected individual	2463	45850	
AR (per million people)	22475	4179	
Severity status			
Mild or moderate case	2053	36850	<0.001 ^a,^*
Severe or critical case	410	9000	
PSCC (%)	16.6	19.6	
Death status			
Non-death case	2454	43426	<0.001 ^a,^*
Death case	9	2424	
CFR (%)	0.4	5.3	

Note: HCW: health care worker; IQR: median and interquartile range; AR: attack rate; PSCC: the proportion of severe and critical cases; CFR: case fatality rate; ^a^ chi-square test; ^b^ Kruskal–Wallis test; * statistically significant.

**Table 2:** Attack rates (per million people) of 36 main hospitals in Wuhan divided by the calendar time interval of a week.

Hospital	23th December–29th December	30th December–5th January	6th January–12th January	13th January–19th January	20th January–26th January	27th January–2nd February	3rd February–9th February	10th February–16th February	17th February–23th February	Total Attack Rate
Hospital1	0	0	0	0	0	0	0	0	0	0
Hospital2	0	0	0	0	0	0	0	0	0	0
Hospital3	0	0	0	0	0	0	0	0	0	0
Hospital4	0	0	0	0	0	0	0	0	0	0
Hospital5	0	0	0	419	419	0	0	419	419	1678
Hospital6	0	0	0	0	0	0	3788	0	0	3788
Hospital7	0	0	238	0	714	714	476	3094	0	5236
Hospital8	485	0	0	0	970	1940	1940	0	0	5335
Hospital9	0	0	0	0	0	0	3394	3394	0	6788
Hospital10	0	0	0	0	3250	1625	1083	1083	542	7584
Hospital11	0	136	136	544	1495	3942	1631	136	0	8021
Hospital12	0	0	516	2581	3614	1033	516	0	0	8260
Hospital13	0	0	0	2188	2188	4376	0	0	0	8753
Hospital14	0	0	649	649	5191	1298	1947	1298	1298	12330
Hospital15	0	0	0	0	1810	3620	3620	4525	0	13575
Hospital16	0	0	0	0	4000	8000	0	4000	0	16000
Hospital17	0	0	1297	0	9079	3891	1297	1297	0	16861
Hospital18	0	0	0	804	804	1608	6431	5627	1608	16881
Hospital19	0	0	0	1319	1319	3958	5277	3958	2639	18470
Hospital20	0	0	1104	4415	4415	11038	0	0	0	20971
Hospital21	0	0	0	2854	8563	13321	3806	951	0	29496
Hospital22	0	0	158	6480	11222	7903	1739	1739	316	29556
Hospital23	0	632	632	6321	10746	6953	1580	5373	316	32554
Hospital24	0	0	0	3155	14196	11041	6309	0	0	34700
Hospital25	0	0	0	7022	5618	11236	5618	8427	0	37921
Hospital26	0	2320	8121	8121	15081	1160	3480	1160	0	39443
Hospital27	0	950	1899	9497	15195	8547	2849	950	0	39886
Hospital28	0	0	0	4773	14320	9547	7160	4773	0	40573
Hospital29	0	0	0	984	11811	9843	14764	2953	984	41339
Hospital30	0	0	0	0	14837	17804	4451	2967	1484	41543
Hospital31	0	0	831	7060	18688	13704	2492	415	0	43189
Hospital32	343	0	1029	5489	19897	11321	3774	1372	0	43225
Hospital33	0	0	774	14706	13158	16254	13158	4644	774	63467
Hospital34	0	0	753	3765	26355	23343	17319	4518	0	76054
Hospital35	0	0	0	1692	43993	38917	6768	8460	1692	101523
Hospital36	0	0	6127	8578	22059	37990	18382	20833	4902	118873

**Table 3:** Risk factors for COVID-19 attack rate of main hospitals in Wuhan by multivariate linear regression.

	RR (95% CI)	*p*-Value
Having fever clinic		
No	Ref	Ref
Yes	1.00 (0.97–1.03)	0.91
Level of hospital		
Provincial	Ref	Ref
Municipal	0.99 (0.97–1.02)	0.65
County-level	1.04 (1.01–1.07)	0.02 *
Type of hospital		
Special	Ref	Ref
General	1.02 (0.99–1.06)	0.11
Chinese medical	1.01 (0.97–1.06)	0.59
Nurse/bed ratio	1.00 (0.98–1.01)	0.63
The county where hospital was located		
Xinzhou	Ref	Ref
Huangpi	1.01 (0.94–1.08)	0.79
Jiangxia	1.01 (0.95–1.08)	0.67
Caidian	1.02 (0.95–1.09)	0.56
Hannan	1.02 (0.95–1.09)	0.63
Dongxihu	1.03 (0.96–1.10)	0.36
Hongshan	1.08 (1.02–1.14)	0.01 *
Qingshan	1.04 (0.98–1.11)	0.14
Wuchang	1.08 (1.03–1.14)	0.01 *
Qiaokou	1.07 (0.99–1.16)	0.08
Hanyang	1.08 (1.01–1.15)	0.03 *
Jiang’an	1.10 (1.03–1.16)	0.003 *
Jianghan	1.07 (1.00–1.16)	0.06

Note: SE: standard error; RR: relative risk; CI: confidence interval; Ref: reference; * statistically significant.

**Table 4:** Risk factors for being severe or critical cases of COVID-19 by logistic regression.

Risk Factors	Crude Data		Univariate Analysis		Multivariate Analysis	
	Mild and Moderate Cases	Severe and Critical Cases	OR (95% CI)	*p*-Value	Adjusted OR (95% CI)	*p*-Value
Age	33 (29, 42) ^a^	37 (30, 45) ^a^	1.02 (0.99–1.05)	0.17	Removed ^c^	NA
Sex						
Female	188 (74.6) ^b^	27 (58.7) ^b^	Ref	Ref	Ref	Ref
Male	64 (25.4) ^b^	19 (41.3) ^b^	2.07 (1.07–3.95)	0.03 *	1.88 (0.92–3.77)	0.08
Days from symptom onset to diagnosis	7 (2, 13) ^a^	7 (5, 11) ^a^	1.01 (0.97–1.05)	0.78	Removed ^c^	NA
Department						
Infection department	171 (67.9) ^b^	15 (32.6) ^b^	Ref	Ref	Ref	Ref
General department	45 (17.9) ^b^	11 (23.9) ^b^	2.79 (1.17–6.46)	0.02 *	2.86 (1.20–6.66)	0.02 *
Ophthalmology department	30 (11.9) ^b^	13 (28.3) ^b^	4.94 (2.12–11.47)	<0.001 *	4.45 (1.88–10.44)	0.001 *
Respiratory department	6 (2.4) ^b^	7 (15.2) ^b^	13.30 (3.96–46.50)	<0.001 *	13.35 (3.93–47.23)	<0.001 *

Note: Age, sex, days from onset to diagnosis and department were included in the multivariate logistic regression, with backward stepwise method for variable selection. OR: odds ratio; CI: confidence interval; NA: not applicable; ^a^ median (IQR); ^b^ count (%); ^c^ removed after the stepwise selection; * statistically significant.
